# Effect of a modified regimen on drug-sensitive retreated pulmonary tuberculosis: A multicenter study in China

**DOI:** 10.3389/fpubh.2023.1039399

**Published:** 2023-01-26

**Authors:** Qiping Ge, Yan Ma, Lijie Zhang, Liping Ma, Caiyan Zhao, Yuhui Chen, Xuerui Huang, Wei Shu, Shengyu Chen, Fei Wang, Bo Li, Xiqin Han, Lian Shi, Xin Wang, Youlun Li, Shangpeng Yang, Wenli Cao, Qianying Liu, Ling Chen, Chao Wu, Bing Ouyang, Furong Wang, Po Li, Xiang Wu, Xiue Xi, Xueyan Leng, Haiqing Zhang, Hua Li, Juan Li, Chengqing Yang, Peng Zhang, Hongzhe Cui, Yuhong Liu, Chengcheng Kong, Zhaogang Sun, Jian Du, Weiwei Gao

**Affiliations:** ^1^Department of Tuberculosis, Beijing Chest Hospital, Capital Medical University, Beijing Tuberculosis and Thoracic Tumor Research Institute, Beijing, China; ^2^Institute of Basic Research in Clinical Medicine, China Academy of Chinese Medical Sciences, Beijing, China; ^3^Institute of Chinese Materia Medica, China Academy of Chinese Medical Sciences, Beijing, China; ^4^Administration Office, Clinical Center on Tuberculosis, China CDC, Beijing, China; ^5^Department of TB Control, Henan Center for Disease Control and Prevention, Zhengzhou, Henan, China; ^6^Department of Tuberculosis, Haerbin Chest Hospital, Haerbin, China; ^7^Department of Outpatients, Center for Tuberculosis Control of Guangdong Province, Guangzhou, Guangdong, China; ^8^Department of Outpatients, Center for Tuberculosis Control of Tianjin, Tianjin, China; ^9^Department of TB Control, Zhejiang Center for Disease Control and Prevention, Hangzhou, Zhejiang, China; ^10^Department of Outpatients, Beijing Center for Disease Control and Prevention, Beijing, China; ^11^Department of Tuberculosis, Shenyang Chest Hospital, Shenyang, Liaoning, China; ^12^Department of TB Control, Heilongjiang Center for Disease Control and Prevention, Haerbin, Heilongjiang, China; ^13^Department of Tuberculosis, The First Affiliated Hospital of Chongqing Medical University, Chongqing, China; ^14^Department of Tuberculosis, Jingzhou Hospital for Infectious Diseases, Jingzhou, Hubei, China; ^15^Department of Infectious Disease, Beijing Geriatric Hospital, Beijing, China; ^16^Department of Tuberculosis, 8th Medical Center, PLA General Hospital, Beijing, China; ^17^Department of Respiratory and Critical Care Medicine, Affiliated Hospital of Zunyi Medical University, Zunyi, Guizhou, China; ^18^Department of Tuberculosis, The 3rd People's Hospital of Zhenjiang, Zhenjiang, Jiangsu, China; ^19^Department of Tuberculosis, Kunming 3rd People's Hospital, Kunming, Yunnan, China; ^20^Department of Medicine, The 4th Hospital of Inner Mongolia Autonomous Region, Huhehaote, China; ^21^Department of Tuberculosis, The 3rd Hospital of Baotou, Baotou, China; ^22^Department of Tuberculosis, Jingmen Center for Disease Control and Prevention, Jingmen, Hubei, China; ^23^Department of Tuberculosis, The First Affiliated Hospital of Xinxiang Medical College, Xinxiang, China; ^24^Department of Tuberculosis, The 3rd Hospital of Qinhuangdao, Qinhuangdao, Hebei, China; ^25^Department of Tuberculosis, Xuzhou Hospital for Infectious Diseases, Xuzhou, Jiangsu, China; ^26^Department of Tuberculosis, Linfen 3rd People's Hospital, Linfen, Shanxi, China; ^27^Department of TB Control, Guangxi Zhuang Autonomous Region Center for Disease Control and Prevention, Nangning, China; ^28^Department of Respiratory and Critical Care Medicine of Wuhan Tuberculosis Institute, Wuhan, Hubei, China; ^29^Department of Tuberculosis, 4th Hospital of Tangshan City, Tangshan, Hebei, China; ^30^Department of Tuberculosis Control, Yanbian Institute of Tuberculosis Prevention and Control, Yanbian, Jilin, China; ^31^Translational Medicine Center, Beijing Tuberculosis and Thoracic Tumor Research Institute, Beijing, China

**Keywords:** pulmonary tuberculosis, retreatment, modified regimen, recurrence, follow-up

## Abstract

**Background and objective:**

Retreatment pulmonary tuberculosis (PTB) still accounts for a large proportion of tuberculosis, and the treatment outcome is unfavorable. The recurrence of retreatment PTB based on long-term follow-up has not been well demonstrated. This study aimed to evaluate effect of a modified regimen on drug-sensitive retreated pulmonary tuberculosis.

**Methods:**

This multicenter cohort study was conducted in 29 hospitals from 23 regions of China from July 1, 2009, to December 31, 2020. Patients were divided into two treatment regimen groups including experimental group [modified regimen (4H-Rt2-E-Z-S(Lfx)/4H-Rt2-E)]and control group [standard regimen (2H-R-E-Z-S/6H-R-E or 3H-R-E-Z/6H-R-E)]. The patients enrolled were followed up of 56 months after successful treatment. We compared the treatment success rate, treatment failure rate, adverse reaction rate, and recurrence rate between two regimens. Multivariate Cox regression model was used to identify the potential risk factors for recurrence after successful treatment with proportional hazards assumptions tested for all variables.

**Results:**

A total of 381 patients with retreatment PTB were enrolled, including 244 (64.0%) in the experimental group and 137 (36.0%) in the control group. Overall, the treatment success rate was significant higher in the experimental group than control group (84.0 vs. 74.5%, *P* = 0.024); no difference was observed in adverse reactions between the two groups (25.8 vs. 21.2%, *P* > 0.05). A total of 307 patients completed the 56 months of follow-up, including 205 with the modified regimen and 102 with the standard regimen. Among these, 10 cases (3.3%) relapsed, including 3 in the experimental group and 7 in the control group (1.5% vs 6.9%, *P* = 0.035). Reduced risks of recurrence were observed in patients treated with the modified regimen compared with the standard regimen, and the adjusted hazard ratio was 0.19 (0.04–0.77).

**Conclusion:**

The modified retreatment regimen had more favorable treatment effects, including higher treatment success rate and lower recurrence rate in patients with retreated drug-sensitive PTB.

## Introduction

Tuberculosis (TB) is the second leading infectious killer after Coronavirus disease 2019 globally and also a major contributor to antimicrobial resistance; the incidence of retreatment pulmonary tuberculosis (PTB) cases was about 392,000 in 2019 (1). In China, Ruan et al. (2) reported that 4.9% of 9,828 people in a retrospective study subsequently developed recurrent TB, and 9.6% were infected with *M. tuberculosis* isolates resistant at least to isoniazid and rifampicin. The first national survey of drug-resistant tuberculosis in China showed that 25.6% of patients with retreated TB had multi-drug resistant (MDR) infections (3). Hence, the retreatment PTB needs more attention and should be addressed.

In some developing countries, the treatment success rate of the standard treatment regimen is also unsatisfactory, with a total cure rate of about 70%; also, the rates are different for different patients (4–6). A multicenter, randomized, parallel, controlled, prospective cohort trial showed that the smear conversion was only 77.6% in the patients with retreatment PTB who adopted the standard retreatment regimen (7). Studies on the regimens on retreatment PTB are ongoing. Recently, the efforts on the replacement of ethambutol with moxifloxacin did not improve the treatment outcomes of retreatment PTB significantly (8), suggesting the difficult task for the ideal regimens for retreatment PTB. The World Health Organization (WHO) has recently updated the guidelines for PTB treatment (9) and recommended that the treatment strategies should be adjusted based on drug susceptibility testing. However, most guidelines were published not for drug-sensitive retreatment PTB but for drug-resistant TB. Therefore, it is necessary to improve the treatment outcome of drug-sensitive PTB. Additionally, the recurrence of retreated PTB after successful treatment has always been a global concern. A study from South Africa reported 14% of recurrence rate after successful treatment with a follow-up of 5 years and 6.8% of recurrence rate with a follow-up of 3 years in China. The relapse of retreated PTB during long-term follow-up after treatment success is limited. The recurrence of PTB brings severe challenges to END TB. Therefore, it is important to ensure the long-term follow-up of patients who have been successfully treated, further understand the recurrence, and identify the relevant factors for recurrence to reduce the recurrence rate. This multicenter bidirectional cohort study was conducted to understand the treatment outcome and recurrence of TB and further provide a reference for clinicians to treat patients with retreated PTB.

## Materials and methods

### Study subjects and settings

Patients with bacteriologically confirmed retreatment PTB having drug susceptibility were retrospectively recruited from 29 TB hospitals and institutions in 23 regions of China from July 1, 2009, to December 31, 2014, with a follow-up by December 31, 2020, who ever attended a bidirectional cohort trial with detailed inclusion and exclusion criteria (Table 1). This study was approved by the ethics committee of the Beijing Chest Hospital affiliated with Capital Medical University (2009–2013). Trial registration: chictr.org Identifier: ChiCTR1800017441 (http://www.chictr.org.cn/historyversionpub.aspx).

**Table 1:** Inclusion and exclusion criteria for patients with retreated TB in the present study.

**Detailed description**	
**Inclusion criteria**	(1) Willing to participate in trial treatment and follow-up; signed informed consent. (2) Aged 18–65 years. (3) Retreatment of pulmonary TB for the first time. (4) Sputum culture-positive patients susceptible to first-line antituberculosis drugs confirmed by the DST. (5) Active pulmonary TB. (6) No obvious abnormalities in liver function, renal function, and blood and urine routine testing. (7) Willing to carry out HIV testing.
**Exclusion criteria**	(1) Resistance to any antituberculosis drug confirmed by DST, including single-drug resistance, poly-drug resistance, and multi-drug resistance. (2) Combined extrapulmonary tuberculosis. (3) HIV antibody positivity. (4) Pregnant or breastfeeding. (5) Severe cardiovascular, liver, kidney, or blood system disease and other serious illnesses. (6) Mental illness. (7) Alcohol abuse. (8) Inability to attend or follow-up treatment. (9) Inability to take oral medications. (10) Allergy or intolerance to any study drug. (11) Participation in another drug clinical trial.

### Study design

The participants in this bidirectional cohort study adopted the modified and standard regimens. The modified retreatment regimen consisted of a 4-month intensive phase, followed a 4-month continuation phase: 4H-Rt_2_-E-Z-S(Lfx)/4H-Rt_2_-E (H, isoniazid (INH); Rt, rifapentine; E, ethambutol; Z, pyrazinamide; S, streptomycin; Lfx, levofloxacin). If S was unavailable, it was replaced with Lfx. In China, the standard treatment regimen consisted of a 2-month intensive phase, followed a 6-month continuation phase: 2H-R-E-Z-S/6H-R-E [R, rifampicin (RIF)]. Patients who could not use S had a 3-month intensive phase: 3H-R-E-Z/6H-R-E. The doses of H and Rt in the modified regimen were increased appropriately compared with the conventional dose in the standard regimen. Table 2 lists the dose and usage of each drug.

**Table 2:** Characteristics of the enrolled patients.

**Characteristic**	**Experimental group**	**Control group**	***P* value**
	***N* (%)**	***N* (%)**	
**Sex**			
Male	189 (77.5)	108 (78.8)	0.756
Female	55 (22.5)	29 (21.2)	
**Age, year**			
18–39	94 (38.5)	49 (35.8)	0.066
40–59	113 (46.3)	77 (56.2)	
≥60	37 (15.2)	11 (8.0)	
**BMI, kg/m** ^2^			0.1157
<18.5	93 (38.1)	40 (29.2)	
18.5–23.9	140 (57.4)	86 (62.8)	
≥24	11 (4.5)	11 (8.0)	
**Retreatment types**			0.236
Relapse	147 (60.3)	90 (65.7)	
Initial treatment failure	32 (13.1)	21 (15.3)	
Unreasonable or irregular antituberculosis treatment for more than 1 month	65 (26.6)	26 (19.0)	
**Background regimen**			
INH (H)^*^ 0.3 g/day	0	137 (100)	
0.4 g/day	244 (100)	0	
Rft (Rt), 0.6 g × 2/week	244 (100)	0	
RIF (R), 0.45–0.6 g/day	0	137 (100)	
EMB (E), 0.75 g/day	244 (100)	137 (100)	
PZA (Z), 1.5 g/day	244 (100)	137 (100)	
Sm (S), 0.75g/day	118 (48.4)	83 (60.6)	
Lfx (F), 0.6g/day	136 (55.7)	0	

Modified regimen, 4H-L_2_-E-Z-S(F)/4H-L_2_-E. Standard regimen, 2H-R-E-Z-S/6H-R-E or 3H-R-E-Z/6H-R-E.

* Based on the body weight (BW), 0.3 g/day (BW <50 kg), and 0.4 g/day (BW ≥50 kg).

In the present study, patients enrolled were divided into two treatment regimen groups including experimental group [adopted the modified regimen (4H-Rt2-E-Z-S(Lfx)/4H-Rt2-E)] and control group [standard regimen (2H-R-E-Z-S/6H-R-E or 3H-R-E-Z/6H-R-E)].

**Figure 1:**
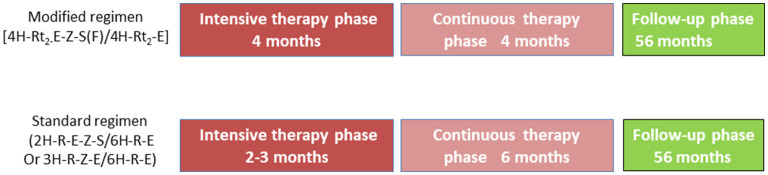
Study design and drug regimens. Patients with drug-sensitive tuberculosis receive modified regimen (4H-L_2−_E-Z-S(F)/4H-L_2_-E) or standard regimen 2H-R-E-Z-S/6H-R-E. H, isoniazid (INH); R, rifampicin (RIF); L, rifapentine (Rft); E, ethambutol (EMB); Z, pyrazinamide (PZA); S, streptomycin (Sm); F, levofloxacin (Lfx).

All the patients with successful treatment were followed up for at least 56 months, and the bacteriological assessment and the evaluation of chest radiological features were performed at least once each year during the follow-up. All patients were told to visit the doctor whenever they felt unwell. Figure 1 shows the flow chart of the study. The primary endpoints included the successful treatment rate at the end of treatment and the recurrence rate after a 56-month follow-up. The secondary endpoints included adverse reactions and treatment failure rate.

### Data collection

A work TB system platform (WTSP) was developed for data collection and management. The information on demography, diagnosis, and treatment was entered in a standard data collection form by two researchers and imported to WTSP after verification. The imaging tests were first reviewed by two physicians and then scanned and uploaded to WTSP. The coordinators contacted the site hospitals for any missing key information, and the monthly online quality assessment and irregular on-site training were conducted during project implementation.

### Assessments

At baseline, the patients provided three sputum samples for sputum smear fluorescence microscopy and culture of *Mycobacterium tuberculosis* in liquid medium using the MGIT 960 method (Becton Dickinson Diagnostic Systems, MD, USA) (10). The morning sputum specimens were obtained at least once every month in the intensive phase and once every 2 months in the continuation phase during treatment. The specimens were processed using the sodium hydroxide and N-acetyl-L-cysteine (NaOH/NALC) method. Mycobacterial speciation was performed using a GenoType Mycobacterium CM kit (Hain Life Sciences, Germany). Drug susceptibility testing (DST) was performed on positive cultures using the MGIT 960 system following the WHO guidelines (11). All tests were performed at the TB reference laboratory, and quality control was routinely performed (12). Besides bacteriological assessment, each patient underwent a physical examination, routine blood and urine tests, liver and renal function tests, and other examinations at each visit. The liver function, renal function, and routine blood tests were also performed once every month. The chest radiological features were obtained before the treatment initiation, at the end of the intensive treatment, and at the end of treatment. All images were evaluated by two physicians and radiologists. The patients received a baseline evaluation and a series of regular safety assessments. The adverse events were recorded daily, and immediately reportable and clinically significant abnormal laboratory results were evaluated as appropriate or as per demand when necessary.

During the follow-up duration, the sputum specimens and chest radiological features were obtained at least once annually. All patients were told to visit the doctor with the symptoms of cough and expectoration for more than 2 weeks accompanied by ineffective anti-inflammatory therapy.

### Definitions and assessments

The treatment outcomes were defined according to the WHO guidelines (13). “Cure” was defined as cases in which the patient completed the treatment according to the program and provided two consecutive negative sputum smear results, including one at the end of the treatment. “Completed treatment” was defined as cases in which the patient had completed the treatment according to the program protocol but did not meet the definition for cure because of the lack of bacteriological results. The “deceased” category included patients who died for any reason during treatment. “Treatment failure” included patients who were sputum smear-positive in the fifth month or later during treatment. “Defaulted” was defined as TB treatment interrupted for two consecutive months or more. “Transfer out” was defined as patients who were transferred to another recording and reporting unit with unknown treatment outcomes. Additionally, the cured and completed treatment categories were classified as “successful treatment,” whereas the others were classified as “unfavorable treatment outcome.” The term TB recurrence used throughout this study denotes a recorded re-diagnosis of TB (as either sputum smear-positive or sputum smear-negative) after “successful” treatment. “Recurrence” was defined as sputum bacteria (smear or culture) positive again during follow-up after successful retreatment. Meanwhile, chest x-ray (or chest computed tomography scanning) showed new lesions or enlarged original lesions, excluding other lung diseases. The time to recurrence was defined as the time between the documented end date of the treatment and the date of re-diagnosis of active TB.

### Statistical analysis

Continuous and categorical variables were presented as medians (interquartile ranges) and percentages (%), respectively. The chi-square test was used for categorical data including demographic and clinical characteristics, and Fisher's exact test was used when the chi-square test was not applicable.

We used the log-rank test to compare the survival curves of time to recurrence after successful treatment in different treatment groups. Further, we used a multivariate Cox regression model to identify the potential risk factors for recurrence after successful treatment with proportional hazards assumptions tested for all variables. Hazard ratios and 95% confidence intervals (CIs) were calculated to demonstrate the risk for recurrence in relation to different treatment regimens. The statistical analysis was conducted using R 3.3.0, and a *P* value < 0.05 indicated a statistically significant difference.

## Results

### Study cohort and characteristics of participants

**Figure 2:**
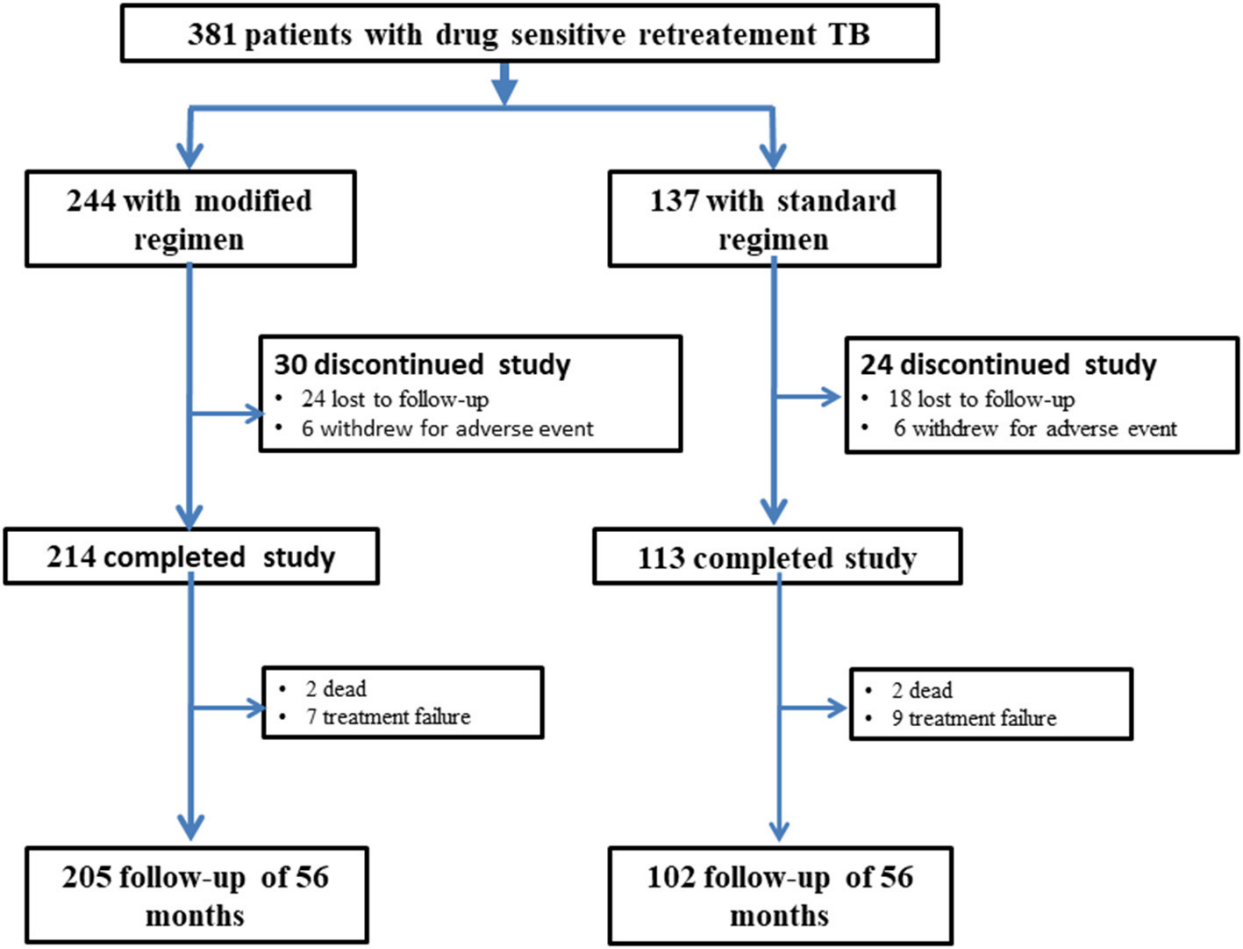
Flow chart of TB subjects enrolled in the study.

Finally, 381 patients were included with 244 and 137 in the experimental and control regimen groups, respectively (Figure 2). No significant differences were found in the demographic or baseline clinical characteristics between the two groups (Table 2).

### Treatment outcomes

**Figure 3:**
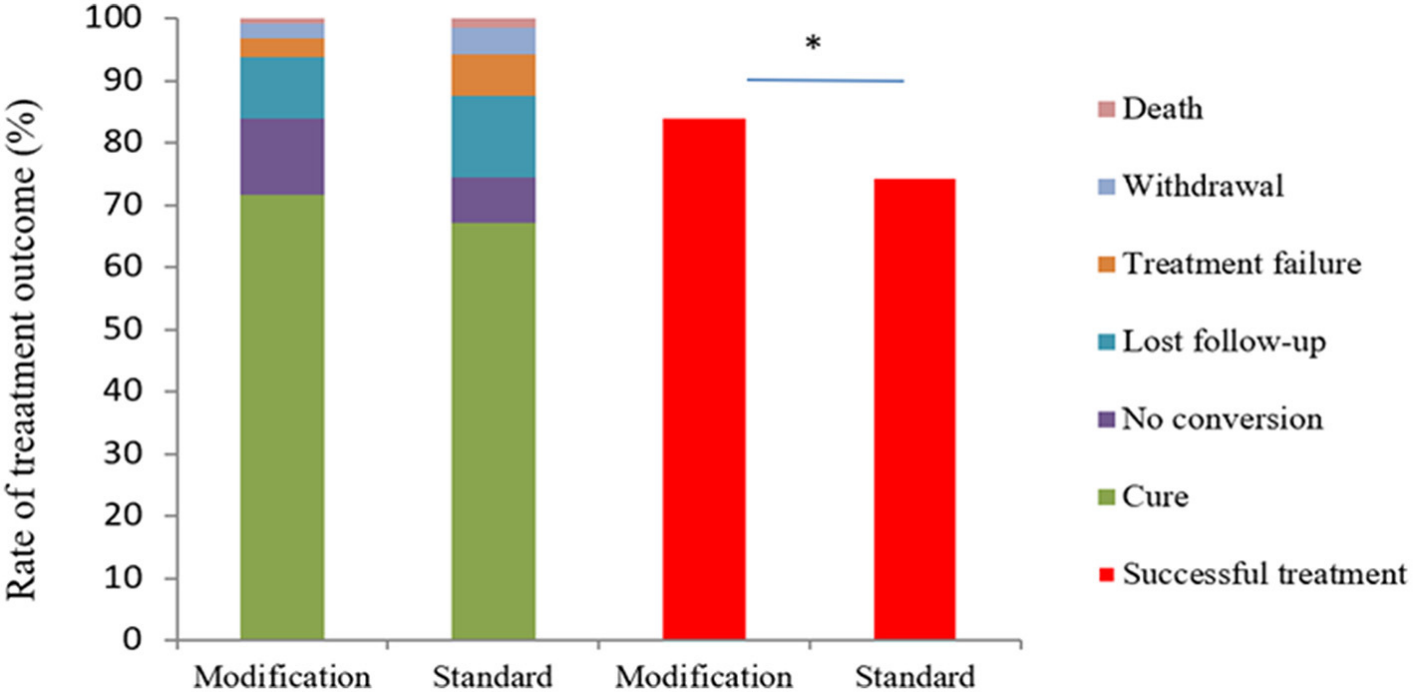
Study outcomes after 8 months according to the protocol-defined analysis and analysis based on the WHO definition. The figure shows study outcomes in the modified intention-to-treat population based on the WHO definitions with respect to study data after 8 months. Patients in the modified regimen group had a higher rate of successful treatment (cure plus no conversion) compared with those in the standard regimen group (χ^2^ = 5.128, *P* = 0.024). ^*^The value of *P* < 0.05 indicate statistical significance.

The treatment success rate was 84.0% (205/244) in the experimental group, which was significantly higher than control group [74.5% (102/137), *P* = 0.024] (Figure 3). The cure and treatment completion rates were 71.7% (175/244) and 12.3% (30/244) in the experimental group, respectively, however, which were 67.2% (92/137) and 7.3% (10/137) in the control group, respectively. The sputum-negative conversion rate at the end of 2-month after treatment was 81.7% (183/224) in the experimental group and 78.4% (69/88) in the control group (χ^2^ = 0.44, *P* = 0.507). There were two deaths because of non-tuberculosis causes in each group. The number of treatment failure cases was seven and nine in the experimental group and control groups, respectively (χ^2^ = 2.986, *P* = 0.084) (Figure 3).

### Adverse reactions

In the experimental group, 63 patients (25.8%) reported 73 adverse reactions, which mainly manifested as gastrointestinal reactions, joint muscle pain, dizziness, tinnitus, and liver function injury (Table 3). Of these, six patients had serious adverse reactions and withdrew from the group, including one patient with drug-induced liver injury, two patients with drug allergy, one patient with headache and tinnitus, one patient with an influenza-like reaction, and one patient with a gastrointestinal reaction.

**Table 3:** Adverse reactions in the studied population^*^.

**Group**	**Abnormal liver function**	**Headache and tinnitus**	**Joint pain**	**Gastrointestinal reactions**	**Skin reaction and others**	**Total**
Experimental group	9 (3.7)	16 (6.6)	17 (7.0)	15 (6.1)	16 (6.6)	73 (29.9)
Control group	8 (5.8)	6 (4.4)	5 (3.6)	8 (5.8)	10 (7.3)	37 (27.0)
*P*	0.329	0.382	0.183	0.904	0.783	0.547

* No significant differences were found between the two groups in any category, as calculated using Fisher's exact test in a *post hoc* analysis.

In the control group, 29 patients (21.2%) reported 37 adverse reactions, which mainly manifested as gastrointestinal reactions, joint pain, dizziness, tinnitus, and skin reactions. Of these, six patients withdrew from the study because of severe adverse reactions involving severe liver injury. No significant difference was found in adverse reactions between the experimental and control groups (χ^2^ = 1.037, *P* = 0.309). Overall, the number of serious adverse reactions, types of reactions, and number of patients with reactions (not including death) were similar between the two study groups during treatment periods.

### Follow-up

**Figure 4:**
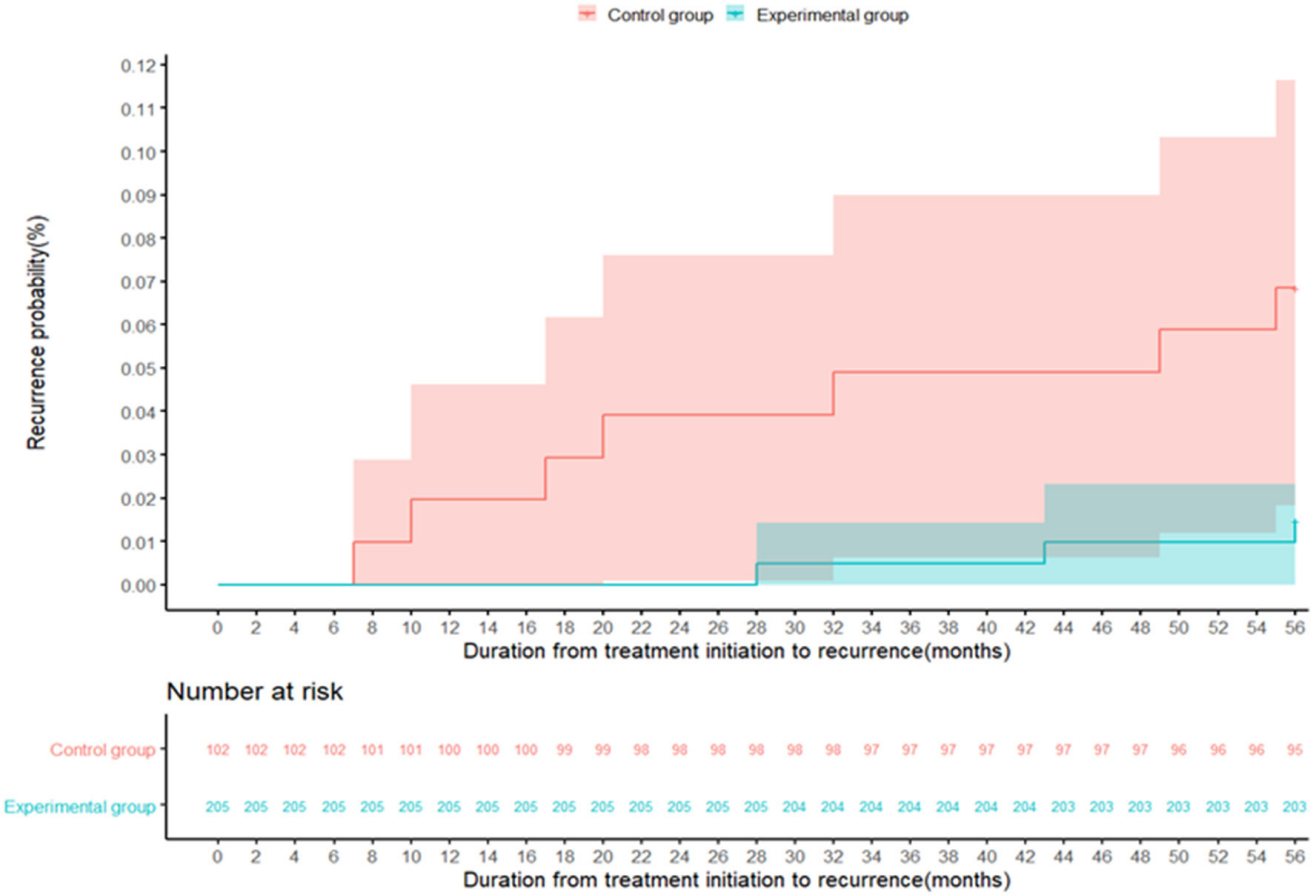
The recurrence probability in the control group (red) and the experimental group (green) during the follow-up period of 56 months. The recurrence of tuberculosis is defined as either a positive M. tb culture or a positive sputum smear fluorescence microscopy during the follow-up.

A total of 307 patients completed the 56-month follow-up, including 205 with the modified regimen and 102 with the standard regimen. Among these, 10 (3.3%) relapsed, including 3 in the experimental group and 7 in the control group (1.5% vs. 6.9%; *P* = 0.035), as shown in Table 4 and Figure 4. Table 4 showed the results of the Cox proportional hazards model for the factors associated with recurrent retreated PTB. The multivariable analysis showed that the modified regimen was associated with a lower risk of recurrence (hazard ratios, 0.19; 95% CI, 0.04–0.77) compared with the standard treatment regimen, after adjusting for age, sex, body mass index (BMI), and comorbidity.

**Table 4:** Factors associated with recurrence after successful treatment.

**Characteristic**	**HR (95% CI)**	***p***
Regimen = Modified regimen	0.19(0.04–0.77)	**0.02**
Age group = 40–59 years^a^	1.5 (0.29–7.81)	0.6
Age group ≥60 years^a^	5.09 (0.74–34.9)	0.1
BMI <18.5^b^	1.85 (0.37–9.21)	0.5
BMI≥24 ^b^	3.38 (0.29–39.8)	0.3
With any comorbidity	2.15 (0.40–11.5)	0.4
Sex = Female	10.221 (0.62–13.6)	>0.9

P-value ≤ 0.05 indicated a statistically significant difference and is presented in boldface in the table. BMI, Body mass index; HR, hazard ratio.

a HRs in comparison with the 18–39 age group.

b HRs in comparison with the 18.5–23.9 BMI group.

## Discussion

The present study showed that the treatment success rate was higher in the experimental group than control group. Moreover, the overall recurrence rate was 3.3% at 56 months after successful treatment, and 1.5 and 6.9% in the experimental and control groups, respectively. We also found that the use of the modified regimen was associated with a lower recurrence rate. The results might provide a reference for clinicians in treating previous PTB patients.

The retreatment PTB is one of the key reasons for the origin of MDR/rifampicin-resistant TB and has imposed great challenges for the TB epidemic, and need to be more attention. Jones-López et al. (14) found that the recommended regimen (category II) yielded unacceptably low treatment response rates in previously treated patients with TB, particularly in subgroups with multidrug-resistant TB and human immunodeficiency virus and was associated with poor long-term outcomes in Kampala, Uganda. In China, the smear conversion of the patients with retreatment PTB who adopted the standard retreatment regimen was only 71.11% as reported 10 years ago (7). In this study, the treatment success rate of the modified regimen (84.0%) increased by nearly 10% and the recurrence rate after the 56-month follow-up decreased by 5.4% compared with the control group.

In this study, the dose of INH was modified in the experimental group based on the patient's body mass instead of the fixed dose of INH (0.3 g/day) in the standard regimen group because the mean body weight of inpatients with PTB increased in decades (15). Appropriately increasing the dose of INH benefits the treatment effect, which has many explanations. First, exposure to lower therapeutic levels of anti-TB drugs is likely to increase drug tolerance and cause the proliferation of resistant strains of *M. tuberculosis* and treatment failure (16, 17), which might be attributed to the increase in the thickness of the cell wall, efflux pump activity, and gene mutations (18). Moreover, the clinical isolates with special genotypes, such as MANU2, has the minimum inhibitory concentration (MIC) to INH between sensitivity and resistance (19). Furthermore, some patients taking INH 0.3 g/day had MIC lower than the target concentration range (20).

Compared with RIF, Rft has a longer half-life and lower effective bacteriostatic concentration (21). Previous studies showed that the Rft doses of 0.6 g/day (22) and 20 mg/(kg · day) (23, 24) showed a trend toward greater efficacy based on the occurrence of culture conversion and did not increase the incidence of adverse events. In this study, we chose Rft at a dose of 0.6 g/day twice a week instead of RIF. Additionally, S had ototoxicity, nephrotoxicity, inconvenient administration, and a high drug resistance rate, while Lfx was much easier to be administered orally with less toxicity to the liver and kidney.

Moreover, a previous study demonstrated that smear positivity after 2 months of treatment for the intensive phase was independently associated with recurrent PTB (2). Nearly 30% of patients were still smear positive at the end of the intensive phase (7). Therefore, in this study, the intensive treatment period was extended from 2–3 months to 4 months to maximize the elimination of rapidly proliferating and most slowly proliferating bacteria.

Recurrent PTB remains a problem in successfully treated patients with sputum smear-positive PTB (2). This study reported a recurrence rate of 1.5% with the modified regimen after the 56-month follow-up, which was much lower than that with the standard regimen. It was also lower than the values reported by studies from India (25) and 12% after a 5-year follow-up in Brazil (26). Notably, the use of the modified regimen reduced the risk of recurrence compared with the standard regimen, which was in line with previous findings (27). However, we also observed that sex, age, BMI, and comorbidity were not associated with the reduced risk of occurrence of recurrent PTB among successfully treated patients with retreated PTB. It suggested that we should pay more attention to the follow-up of patients with retreated PTB receiving different regimens after successful treatment.

## Data availability statement

The original contributions presented in the study are included in the article; further inquiries can be directed to the corresponding authors.

## Ethics statement

The studies involving human participants were reviewed and approved by the Chinese Clinical Trial Registry (ChiCTR1800017441) and the Ethics Committee of Beijing Chest Hospital, Capital Medical University. The patients/participants provided their written informed consent to participate in this study.

## Author contributions

QG: conceptualization, investigation, visualization, and writing—original draft. YM and LZ: investigation, visualization, and writing—original draft. LM: investigation. CZ, YC, XHu, SC, FeW, BL, XHa, LS, XWa, YL, SY, WC, QL, LC, CW, BO, FuW, PL, XWu, XX, XL, HZ, HL, JL, CY, PZ, HC, and CK: patient inclusion, follow-up, and data entry. WS and YL: methodology, software, and formal analysis. ZS, JD, and WG: review and editing. All authors contributed to the article and approved the submitted version.
